# PPARδ agonist protects against osteoarthritis by activating AKT/mTOR signaling pathway-mediated autophagy

**DOI:** 10.3389/fphar.2024.1336282

**Published:** 2024-03-21

**Authors:** Guantong Sun, Xiaodong Li, Pengcheng Liu, Yao Wang, Cheng Yang, Shuhong Zhang, Lei Wang, Xiaoqing Wang

**Affiliations:** ^1^ Department of Orthopedics, Shanghai Ninth People’s Hospital, Shanghai Jiao Tong University School of Medicine, Shanghai, China; ^2^ Department of Orthopedics, Shanghai General Hospital, Shanghai Jiao Tong University, Shanghai, China

**Keywords:** PPARδ, osteoarthritis, autophagy, apoptosis, AKT/mTOR signaling

## Abstract

Osteoarthritis (OA) is the most prevalent degenerative joint disease, and PPARs are involved in its pathogenesis; however, the specific mechanisms by which changes in PPARδ impact the OA pathogenesis yet to be discovered. The purpose of this study was to ascertain how PPARδ affects the onset and development of OA. *In vitro*, we found that PPARδ activation ameliorated apoptosis and extracellular matrix (ECM) degradation in OA chondrocytes stimulated by IL-1β. In addition, PPARδ activation may modulate AKT/mTOR signaling to partially regulate chondrocyte autophagy and apoptosis. *In vivo*, injection of PPARδ agonist into the articular cavity improved ECM degradation, apoptosis and autophagy in rats OA models generated by destabilization medial meniscus (DMM), eventually delayed degeneration of articular cartilage. Thus, targeting PPARδ for OA treatment may be a possibility.

## 1 Introduction

Osteoarthritis (OA) is a chronic degenerative osteoarthritic joint disease, which is defined by the development of bone redundancy, subchondral bone sclerosis, and progressive and irreversible degeneration of articular cartilage, mostly affecting adults in their middle and later years (Creamer and Hochberg, 1997; Wood et al., 2023). The sole type of cells found in articular cartilage are chondrocytes, which has a low regenerative capacity, but they are essential in preserving the cartilage tissue’s homeostasis. OA is molecularly characterized by the degradation and metabolic disturbance of ECM, mainly in the form of decreased collagen synthesis and overexpression of matrix metalloproteinases (Glyn-Jones et al., 2015). In addition, multiple cell death mechanisms, including oxidative stress, apoptosis, and cell autophagy, impair chondrocytes’ ability to operate normally (Rahmati et al., 2017; Ansari et al., 2020). Today, OA remains difficult to treat and musculoskeletal disorders, including OA, are the leading cause of disability in the global elderly population (Rahmati et al., 2017; Abramoff and Caldera, 2020). Therefore, in order to develop novel therapeutic methods in the field of osteoarthritis research, a deeper comprehension of the precise molecular pathways of apoptosis and ECM degradation in chondrocytes is required.

Autophagy is a cellular self-defense mechanism, removing damaged proteins and organelles to preserve intracellular homeostasis against apoptosis (Glick et al., 2010; Guan et al., 2013; Kim and Lee, 2014). One of the main factors causing OA to advance is autophagy impairment (Zheng et al., 2021). In mice articular cartilage, deletion of ATG5 increases chondrocytes apoptosis and accelerates the development of OA (Bouderlique et al., 2016). Furthermore, HECTD1 modulates autophagy of chondrocytes through regulating Rubicon’s degradation and ubiquitination, thereby having a beneficial influence on OA (Liao et al., 2023). In conclusion, autophagy activation might be a useful tactic for treating OA.

Peroxisome Proliferator-Activated Receptors (PPARs) are recognized as nuclear receptor proteins that are implicated in inflammation, lipid metabolism, cell division and proliferation, and the preservation of the body’s energy metabolism equilibrium (Michalik and Wahli, 2006; Mirza et al., 2019; Christofides et al., 2021). Currently, three distinct PPARα, PPARβ/δ, and PPARγ subtypes in mammals have been discovered (Dubois et al., 2017). Multiple human disorders, including cancer (Muller, 2017; Wagner and Wagner, 2020), diabetes (Gross et al., 2017), autoimmune diseases (SLE) (Liu et al., 2020), and hypertension-induced renal fibrosis (Corrales et al., 2018), have been identified to be related with PPARs. In a model of diabetic cardiomyopathy in mice, PPARδ attenuated endoplasmic reticulum stress by upregulating autophagy (Palomer et al., 2014). In addition, PPARδ activation led to the renewal of type II hyaline cartilage and the healing of bone defects following the implantation of mesenchymal stem cells with PPARδ agonists (Song et al., 2022). PPARδ agonist also significantly increased MSC chondrogenesis and glycosaminoglycan and collagen II expression in chondrocytes produced from MSCs (Heck et al., 2017). The aforementioned research indicates that PPARδ is linked to inflammation, and cartilage regeneration. Furthermore, PPARα activation increases the autophagic fluxes of chondrocytes (Zhou et al., 2019). Deficiency of PPARγ in mouse articular chondrocytes led to upregulation of articular cartilage mTOR thereby inhibiting autophagy, resulting in increased chondrocyte apoptosis, which in turn accelerated OA progression (Vasheghani et al., 2015). However, it is unknown if PPARδ influences autophagy during the advancement of OA.

In this investigation, we explored PPARδ expression and function within OA. We found that PPARδ expression was downregulated in OA rats articular cartilage and OA chondrocytes stimulated by IL-1β. Subsequently, we further investigated the specific mechanisms by which PPARδ regulates OA and found that PPARδ improved ECM degradation and attenuated chondrocyte apoptosis via autophagy mediated by the AKT/mTOR signaling, which provided defense against cartilage deterioration. Therefore, our research implies that PPARδ could potentially be used as a molecular target for OA treatment.

## 2 Materials and methods

### 2.1 Reagents

Recombinant rat IL-1β (Peprotech), PPARδ (abcam, ab23673), Aggrecan (abcam, ab3773), Collagen II (abcam, ab239007), MMP13 (abcam, ab39012), SOX9 (abcam, ab185966), Cleaved-PARP (Cell Signaling Technology, Asp214), Cleaved-caspase3 (Cell Signaling Technology, Asp175), Bcl-2 (abcam, ab194583), Bax (Cell Signaling Technology D2E11), ATG5 (abcam, ab108327), Beclin1 (abcam, ab62557), LC3 (Sigma-Aldrich, L8918), GW501516 (Selleck, S5616), GSK3787 (Selleck, S8025).

### 2.2 Isolation and culture of rat primary chondrocytes

Male SD rats that were 4 weeks old were euthanized, and the articular cartilage of rats femoral head was stripped under aseptic conditions, and subsequently cut into 1-cubic-millimeter pieces, rinsed with PBS 3 times, and placed in culture dishes containing 0.2% type II collagenase and incubated in an incubator containing 5% CO_2_ and 37°C for 8 h. Digested cartilage was collected, centrifuged for 5 min, then pour off the liquid above the cell precipitate. After being resuspended in DMEM/F12, the cells were cultured in cell incubator. After then, every two to 3 days, the cell medium was replaced, and 0.25% EDTA was used to digest the cells when they reached 80%–90% density, subsequently moved to 10 cm dishes. The morphology of chondrocytes did not significantly alter between generation P0 and P2; therefore, in all cell experiments, second-generation chondrocytes were used.

### 2.3 Chondrocyte treatment

Rat chondrocytes were treated to different amounts of IL-1β (0, 10, 20, 30 ng/mL) for 24 h, and IL-1β (10 ng/mL) for different durations (0, 12, 24, 48 h) in order to examine the expression of PPARδ in these cells. In PPARδ′s *in vitro* functional investigation, chondrocytes were pre-exposed to PPARδ agonist (GW501516, 100 nM) (Poleni et al., 2007) and PPARδ inhibitor (GSK3787, 1 μM) (Shearer et al., 2010), subsequently treated for 24 h with IL-1β (10 ng/mL). To further explore the specific molecular mechanisms involved in PPARδ mediating OA, we assessed whether PPARδ attenuates OA through AKT/mTOR-regulated autophagy by pre-treating chondrocytes with PPARδ agonist (GW501516, 100 nM) and PPARδ inhibitor (GSK3787, 1 μM), and then treating cells with IL-1β (10 ng/mL) for 24 h. Dimethyl sulfoxide (DMSO) was used to dissolve GW501516 and GSK3787, and the final concentration of it in cell culture medium was less than 0.1%.

### 2.4 Western blot

Pre-cooled PBS was added to the 6-well plate, and the adherent chondrocytes were washed by gently shaking 3 times, and then RIPA buffer containing 10% phosphatase inhibitor and protease inhibitor (Roche Diagnostics, Basel, Switzerland) was added to each well; after that total protein was extracted from the chondrocytes. Subsequently, we measured protein concentration, utilizing the experimental tool BCA assay kit. SDS-PAGE gels was used to separate equal amounts of protein (15–20 μg), and then transferred onto polyvinylidene fluoride (PVDF) membrane measuring 0.22 µm. After that, the PVDF membrane was placed in the configured 5% non-fat milk, and then incubated on a horizontal shaker with shaking. After sealing was completed, the blocked membrane was placed in TBST and then allowed to be washed by shaking on a horizontal shaker for a total of three times. The membranes were then incubated sequentially with primary antibody (4°C, overnight) and secondary antibody (1 h). Finally, the membrane was detected employing the Odyssey image scanner (Li-COR. Inc., Lincoln, NE, United States). ImageJ was used to quantify the gray value of blots.

### 2.5 Quantitative real-time PCR

TRIzol^®^ reagent (Invitrogen, Waltham, MA, United States) was used to extract total RNA from the treated chondrocytes. Using reverse transcription reagent (TaKaRa Bio Inc., Kusatsu, Shiga, Japan), cDNA was produced from RNA samples and then the mRNA expression of each gene was determined with qRT-PCR. The target genes’ expression levels were determined using the 2^−ΔΔCT^ method, normalized with GAPDH. Table 1 contains a list of all primer sequences used in this work.

**TABLE 1:** Primer sequences for qRT-PCR.

Gene	Species	Forward primer	Reverse primer
PPARδ	Rat	AAA​CCC​ACG​GTA​AAG​GCG​G	CTG​TTC​CAT​GAC​TGA​CCC​CC
Aggrecan	Rat	CCT​CTC​AAG​CCC​TTG​TCT​GAA​T	ACA​TTG​CTC​CTG​GTC​GAT​CTC​A
Collagen II	Rat	GAT​GTA​TGG​AAG​CCC​TCG​TCC	CCT​TTG​GCC​CTA​ATT​TTC​CAC​T
MMP13	Rat	GGG​AAC​CAC​GTG​TGG​AGT​TAT	GAC​AGC​ATC​TAC​TTT​GTC​GCC
SOX9	Rat	TCG​GTG​AAG​AAT​GGG​CAA​GC	GAC​CCT​GAG​ATT​GCC​CGG​AG
ATG5	Rat	CAC​TGG​GAC​TTC​TGC​TCC​TG	AAC​CAA​GCC​AAA​CCG​AGG​TG
Beclin1	Rat	CTC​GTC​AAG​GCG​TCA​CTT​CT	TAG​ACC​CCT​CCA​TTC​CTC​AG
LC3	Rat	GCC​GGA​GCT​TCG​AAC​AAA​GA	CAG​CTG​CTT​CTC​ACC​CTT​GT
GAPDH	Rat	CTC​TCT​GCT​CCT​CCC​TGT​TC	CGA​TAC​GGC​CAA​ATC​CGT​TC

### 2.6 Immunofluorescence

Plates with six wells were used to cultivate chondrocytes. The corresponding subgroups were first pretreated with GW501516 and GSK3787, followed by the addition of IL-1β (10 ng/mL, 24 h). After being treated with paraformaldehyde (4%), chondrocytes underwent three PBS washes. After that, chondrocytes were treated in Triton X-100, and three PBS washes afterwards. Then they were closed for 1 h with 5% FBS, and three PBS washes afterwards. The configured primary antibody was then covered with chondrocytes (4°C, overnight), three PBS washes afterwards. After incubating with fluorescent antibody (ab203438), chondrocytes were placed in a dark room (1 h). After that, chondrocytes underwent DAPI treatment (5 min) and were placed in a dark room, and three PBS washes. The residual PBS on the slides was then blotted with absorbent paper or air-dried. Finally, anti-fluorescence quenching tablet was used to seal chondrocytes and imaged by confocal microscopy (Leica Microsystems GmbH).

### 2.7 TUNEL

The corresponding subgroups were first pretreated with GW501516 and GSK3787, followed by the addition of IL-1β (10 ng/mL, 24 h). After being treated with paraformaldehyde (4%, 30 min), chondrocytes underwent three PBS washes. Finally, TUNEL staining was performed using (TUNEL Apoptosis Assay Kit, Beyotime). The laser confocal microscope (Leica Microsystems GmbH) was used to take all of the images.

### 2.8 Animal models

A total of twenty-four SD rats, aged 8 weeks, were obtained for the present study (Shanghai Bikay Koyi Biotechnology Co., Ltd., Shanghai, China). Briefly, the sample size for animal experiments was selected according to a well-designed experimental program, which was also authorized by Shanghai Ninth People’s Hospitals’ Ethics Committee (SH9H-2023-A834-1), and the OA rat model was established by DMM surgery as previously reported. Four groups were randomly assigned to these rats: Sham, DMM, DMM + DMSO, and DMM + GW501516. After anesthesia, the rats’ capsule was sliced medially to the patellar tendon, and microsurgical scissors were used to sever the medial meniscal ligament and meniscus. Rats in Sham also received arthrotomy, but the medial meniscal ligament was not removed. After DMM, the DMM + GW501516 group was injected intra-articularly with GW501516 dissolved in DMSO (10 mg/kg) twice a week. The same volume of DMSO was intra-articularly injected into the DMM + DMSO group. After surgery, they were placed in cages where they could move freely. The knee joints of the executed rats were removed after 8 weeks, followed by histological analysis.

### 2.9 Histopathologic analysis

The knee was fixed using formalin, followed by decalcification with EDTA (10%, 2 weeks), followed by dehydration and embedding in paraffin. Histological sections (4–6 μm) were performed, then came H&E and Safranin O staining. Finally, the OARSI score was chosen to evaluate the severity of damage to cartilage.

### 2.10 Immunohistochemical staining

The embedded paraffin sections were dewaxed and then closed with hydrogen peroxide (H_2_O_2_, 3%). After that, primary antibodies were added to the tissue sections and incubated overnight at 4°C. The tissue sections were then incubated for 30 min with the secondary antibodies. Finally, in order to provide an immunohistochemistry staining signal, the tissue sections stained with 3,3-diaminobenzidine. ImageJ was used to count positively stained cells.

### 2.11 Micro-CT

After isolation of the knee specimen, the soft tissue in the vicinity was excised, and knee specimen was fixed for 48 h with 4% PFA. The knee joint was scanned using micro-CT followed by 3D reconstruction.

### 2.12 Statistical analysis

Data were reported as mean ± SD. An unpaired Student’s t-test was used to assess the differences between the two groups. Differences between three or more groups were made using one-way analysis of variance (ANOVA) and Tukey’s *post hoc* test. Ranked data was analyzed using the Kruskal–Wallis H test. For all analyses of statistics, GraphPad Prism 9.0 was utilized. *p < 0.05.

## 3 Results

### 3.1 PPARδ expression is decreased in IL-1β-induced OA chondrocytes

**FIGURE 1:**
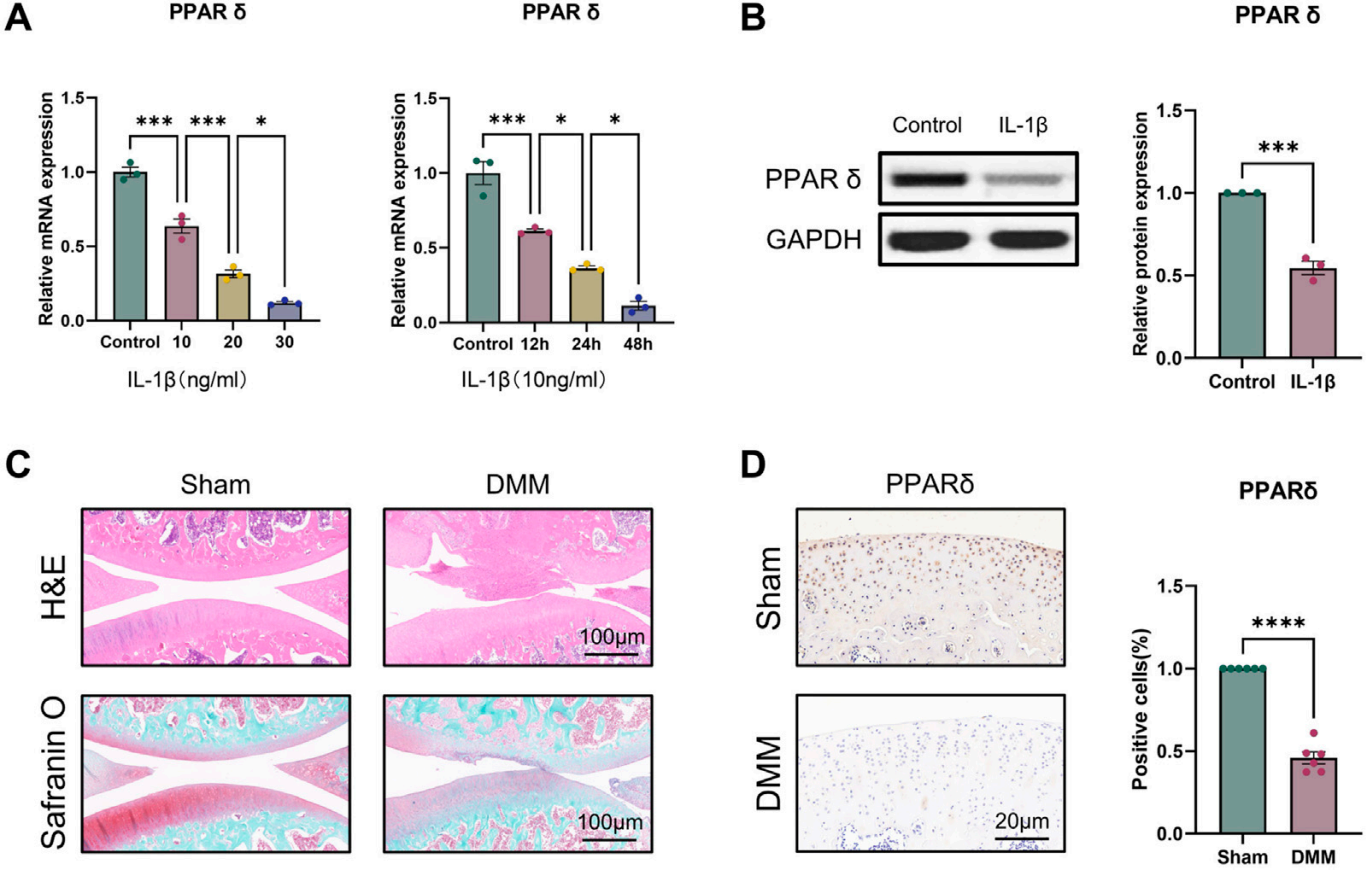
PPARδ expression is decreased in IL-1β-induced OA chondrocytes. **(A)** Quantitative real-time PCR assessment of PPARδ expression after treatment of rat primary chondrocytes with different concentrations of IL-1β at different time points. (*n* = 3) *p < 0.05, ***p < 0.001. **(B)** The expression of PPARδ in rat chondrocytes was assessed using Western blot. (*n* = 3) ***p < 0.001. **(C)** Safranin O staining, H&E staining, and **(D)** immunohistochemical staining of rat knee joints. (*n* = 6) ****p < 0.001.

To evaluate the potential function of PPARδ in OA, we first investigated the expression of PPARδ *in vitro*. qRT-PCR results showed that, compared to controls, either treated with 10 ng/mL IL-1β for different times or different concentrations of IL-1β for 24 h, PPARδ was significantly reduced (Figure 1A). We also found that PPARδ expression was similarly markedly downregulated in OA chondrocytes by Western blot (Figure 1B). Furthermore, we also investigated PPARα/γ expression in rat chondrocytes by Western blot. Following IL-1β treatment, PPARα and PPARγ expression dropped in comparison to control, although the decline was not as significant as that of PPARδ (Supplementary Figure S1). To further validate the above findings and explore the expression of PPARδ *in vivo*, we used DMM to establish rat OA model. Compared to rats in sham, we observed the decreased proteoglycans (red) in OA rats, indicating cartilage degeneration (Figure 1C). Furthermore, compared with the Sham, in the articular cartilage of the DMM rats, we discovered that PPARδ expression was considerably decreased (Figure 1D). Therefore, we conclude that PPARδ expression is markedly decreased during the development of OA.

### 3.2 PPARδ regulates IL-1β-induced degradation of cartilage ECM

**FIGURE 2:**
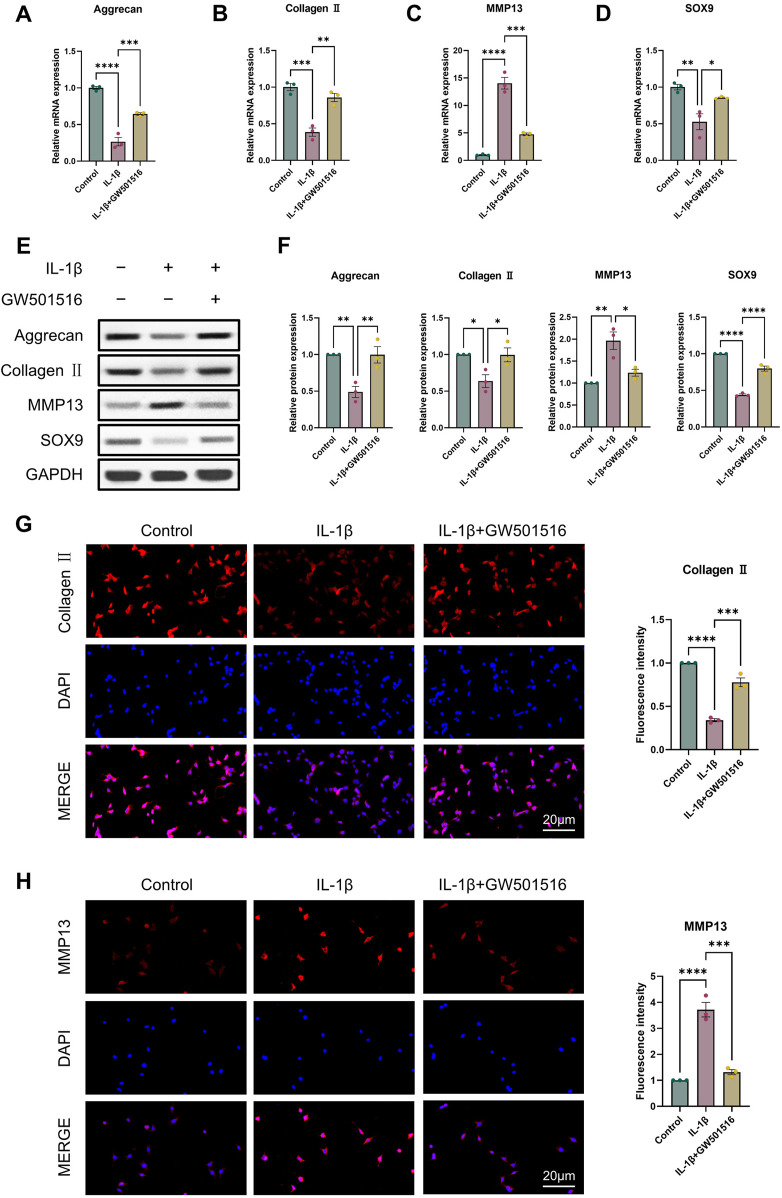
Activation of PPARδ inhibits IL-1β-induced cartilage ECM degradation. Chondrocytes were treated with PPARδ agonist (GW501516) and/or IL-1β. **(A–D)** Quantitative real-time PCR analysis of Aggrecan, Collagen II, MMP13 and SOX9 in rat chondrocytes using GAPDH as an endogenous control. **(E, F)** Protein expression of Aggrecan, Collagen II, MMP13, SOX9 in rat articular chondrocytes was assessed by Western blot with GAPDH as an endogenous reference and its quantification by ImageJ. **(G, H)** Immunofluorescence staining results of Collagen II and MMP13 in rat chondrocytes (bar:20 μm). All data are expressed as mean ± SD (*n* = 3); *p < 0.05, **p < 0.01, ***p < 0.001, ****p < 0.0001.

**FIGURE 3:**
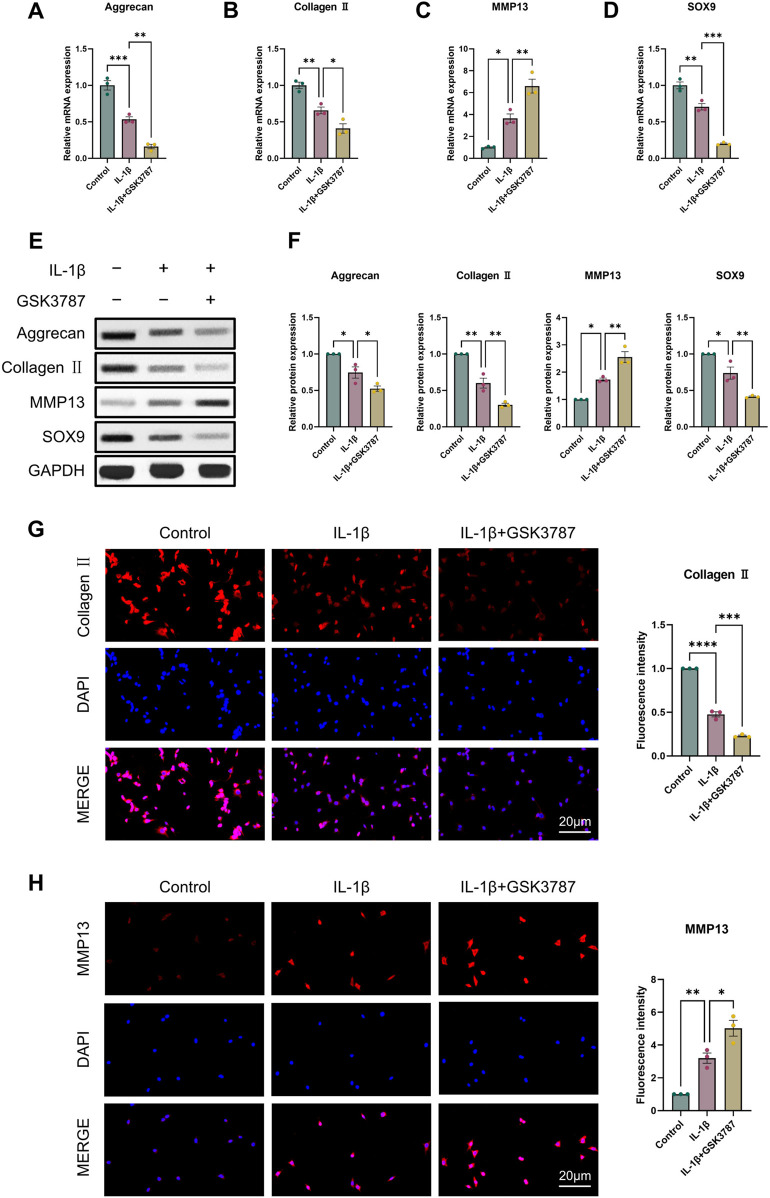
Antagonizing PPARδ activation promotes IL-1β-induced cartilage ECM degradation. Chondrocytes were treated with PPARδ antagonist (GSK3787) and/or IL-1β. **(A–D)** Quantitative real-time PCR analysis of Aggrecan, Collagen II, MMP13 and SOX9 in rat chondrocytes using GAPDH as an endogenous control. **(E, F)** Protein expression of Aggrecan, Collagen II, MMP13, SOX9 in rat articular chondrocytes was assessed by Western blot with GAPDH as an endogenous reference and its quantification by ImageJ. **(G, H)** Immunofluorescence staining results of Collagen II and MMP13 in rat chondrocytes (bar:20 μm). All data are expressed as mean ± SD (*n* = 3); *p < 0.05, **p < 0.01, ***p < 0.001, ****p < 0.0001.

For the reason to examine PPARδ′s impact on OA in more detail, we isolated rat chondrocytes and assessed Aggrecan, Collagen II, SOX9 and MMP13 expression by Western blot. Our results showed that PPARδ activation reduced MMP13 expression, and increased Aggrecan, Collagen II, and SOX9 genes’ expression (Figures 2A–F). Immunofluorescence staining results also confirmed this conclusion (Figures 2G, H). In contrast, Aggrecan, Collagen II and SOX9 expression was significantly decreased after PPARδ antagonism, while MMP13 expression was significantly increased (Figure 3). Taken together, these findings suggest that PPARδ activation may inhibit articular cartilage degeneration in OA.

### 3.3 PPARδ regulates IL-1β-induced chondrocyte apoptosis

**FIGURE 4:**
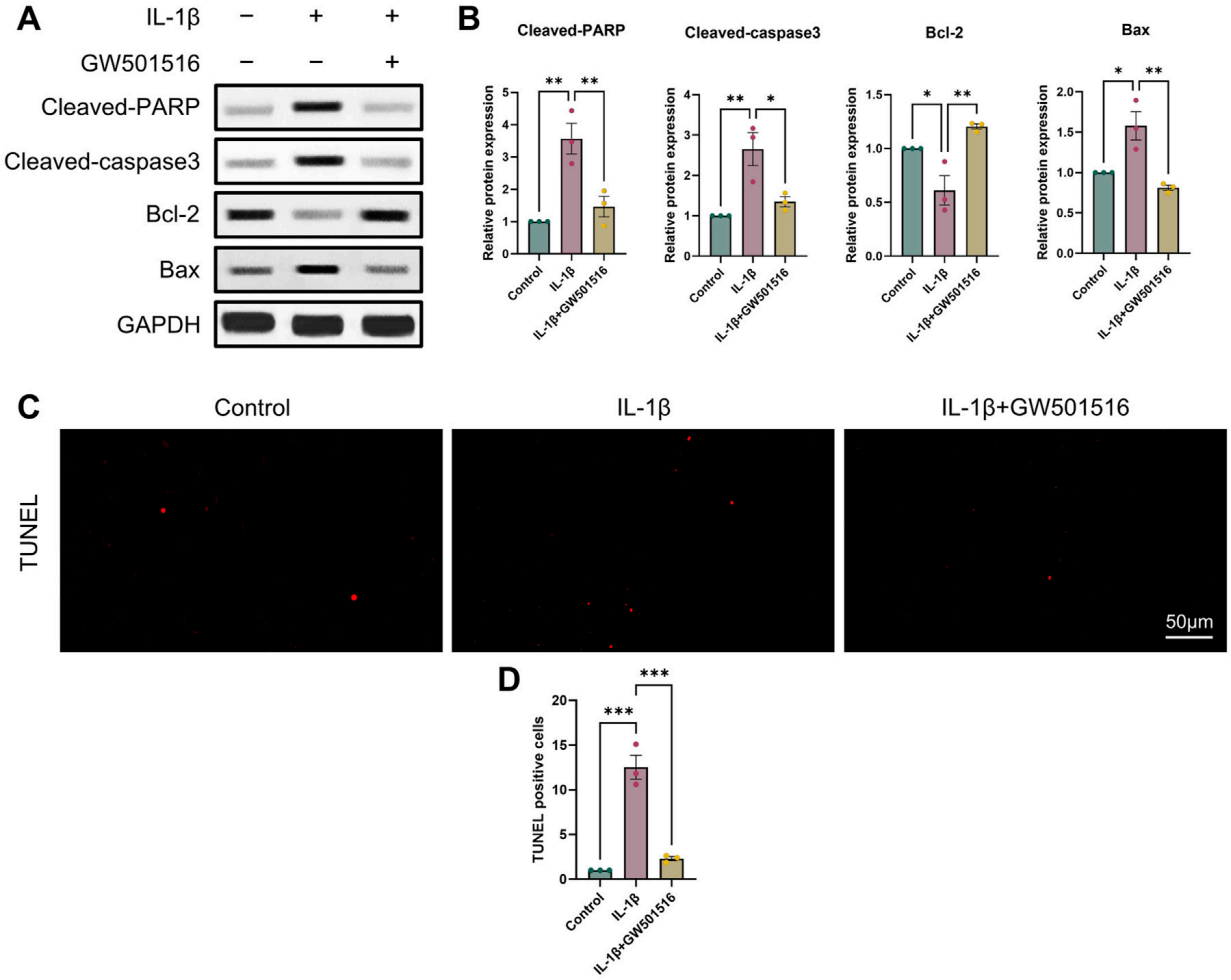
Activation of PPARδ inhibits IL-1β-induced chondrocyte apoptosis **(A, B)** Western blot and quantification of Cleaved-PARP, Cleaved-caspase3, Bcl-2, Bax expression in rat chondrocytes exposed to IL-1β or PPARδ agonist (GW501516) using ImageJ. **(C, D)** Chondrocyte apoptosis was assessed using TUNEL staining (bar:50 μm). All data are expressed as mean ± SD (*n* = 3); *p < 0.05, **p < 0.01, ***p < 0.001.

**FIGURE 5:**
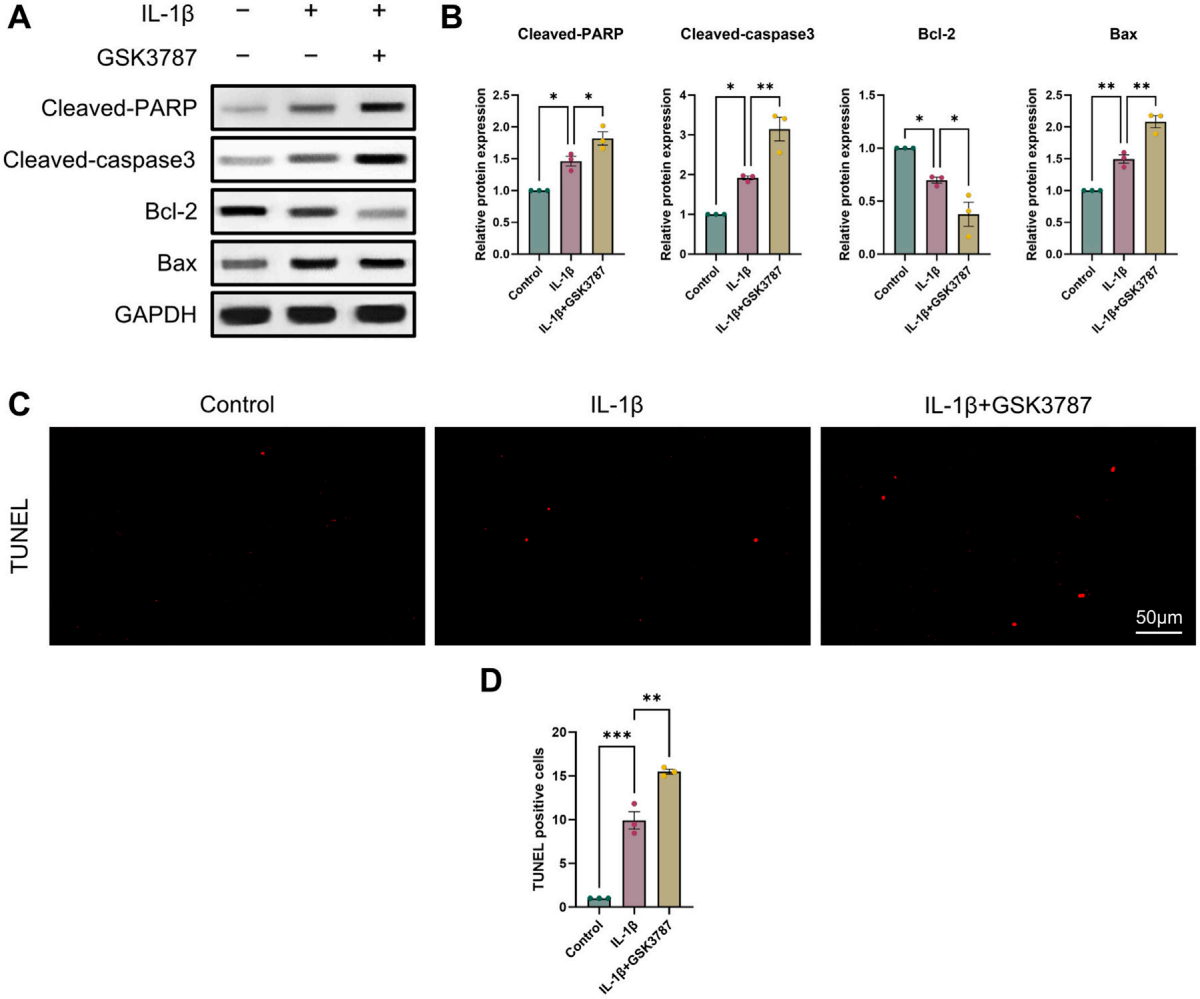
Antagonizing PPARδ activation promotes IL-1β-induced chondrocyte apoptosis. **(A, B)** Western blot and quantification of Cleaved-PARP, Cleaved-caspase3, Bcl-2, Bax expression in rat chondrocytes exposed to IL-1β or PPARδ antagonist (GSK3787) using ImageJ. **(C, D)** Chondrocyte apoptosis was assessed using TUNEL staining (bar:50 μm). All data are expressed as mean ± SD (*n* = 3); *p < 0.05, **p < 0.01, ***p < 0.001.

It is commonly established that OA and chondrocyte apoptosis are tightly related (Hosseinzadeh et al., 2016). To learn more about how PPARδ affects chondrocyte apoptosis, we assessed Cleaved-PARP, Cleaved-caspase3, Bcl-2, and Bax expression following PPARδ activation and antagonism. The results demonstrated that PPARδ activation might reverse the considerable decrease of Bcl-2 expression and the significant increase of Cleaved-PARP, Cleaved-caspase3, and Bax expression that IL-1β had caused. TUNEL staining also confirmed the above results (Figures 4C, D and Supplementary Figure S2A). In addition, we found that PPARδ antagonism in chondrocytes further resulted in the significant increase in Cleaved-PARP, Cleaved-caspase3 and Bax expression as well as the significant decrease in Bcl-2 expression (Figures 5A, B). Similarly TUNEL staining also yielded the same results (Figures 5C, D and Supplementary Figure S2B). Thus, we found that PPARδ activation could rescue chondrocyte apoptosis.

### 3.4 PPARδ regulates IL-1β-induced chondrocyte autophagy

**FIGURE 6:**
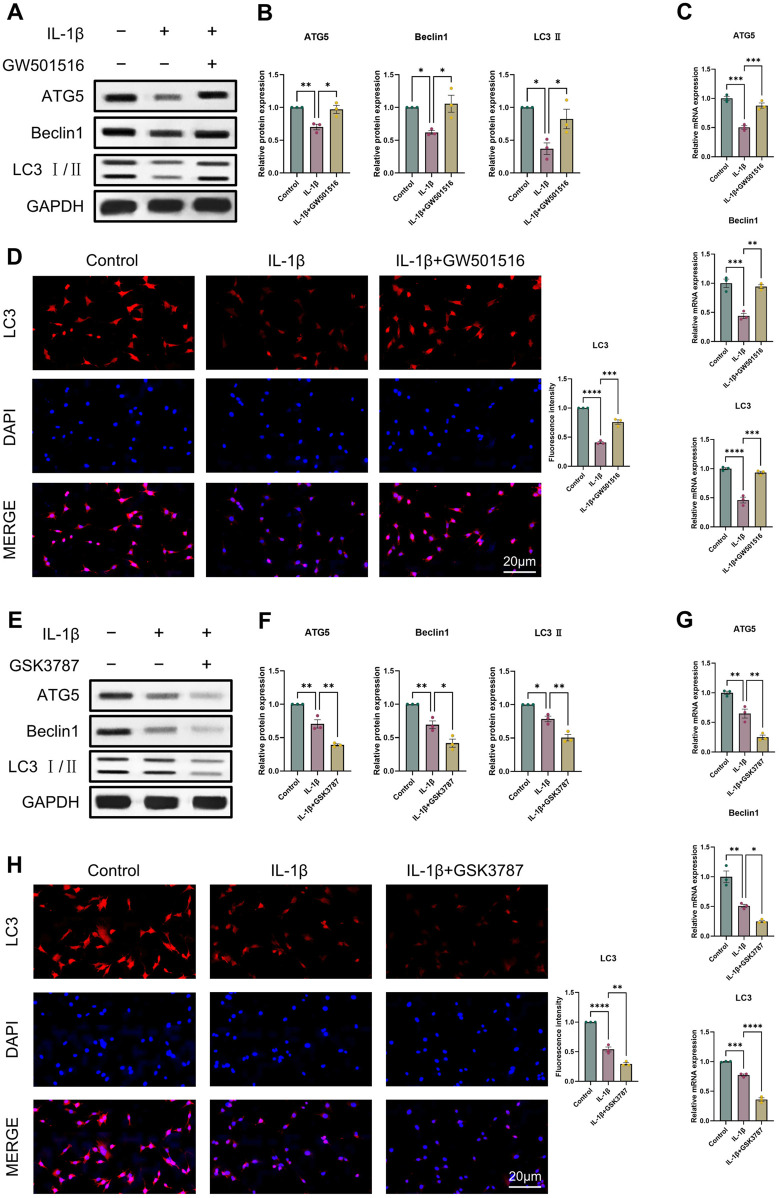
PPARδ regulates IL-1β-induced chondrocyte autophagy. **(A, B)** Western blot and quantification of ATG5, Beclin1 and LC3 Ⅱ expression in rat chondrocytes using ImageJ. **(C)** Quantitative real-time PCR analysis of ATG5, Beclin1 and LC3 in rat chondrocytes using GAPDH as an endogenous control. **(D)** Immunofluorescence staining results of LC3 in rat chondrocytes (bar:20 μm). Rat chondrocytes were pre-exposed to GSK3787 and subsequently treated with IL-1β (10 ng/mL) for 24 h. **(E, F)** Western blot and quantification of ATG5, Beclin1 and LC3 Ⅱ expression in rat chondrocytes using ImageJ. **(G)** Quantitative real-time PCR analysis of ATG5, Beclin1 and LC3 in rat chondrocytes using GAPDH as an endogenous control. **(H)** Immunofluorescence staining results of LC3 in rat chondrocytes (bar:20 μm). All data are expressed as mean ± SD (*n* = 3); *p < 0.05, **p < 0.01, ***p < 0.001, ****p < 0.0001.

It was discovered that OA and reduced autophagy are closely related. Thus, we first examined ATG5, Beclin1, LC3 II expression using Western blot and qRT-PCR to assess how PPARδ activation and antagonism affect chondrocyte autophagy. Results indicated that PPARδ activation reversed the IL-1β-induced impaired autophagy and upregulated ATG5, Beclin1, and LC3 II expression (Figures 6A–C). Furthermore, we also confirmed the rescue effect of PPARδ upon autophagic flux by immunofluorescence staining (Figure 6D). In contrast, PPARδ antagonism further enhanced the IL-1β-induced impaired autophagy (Figures 6E–H). These results suggest that PPARδ might rescue the IL-1β-induced impaired autophagy.

### 3.5 PPARδ activation increases the expression of genes associated to autophagy and reduces apoptosis in chondrocytes

**FIGURE 7:**
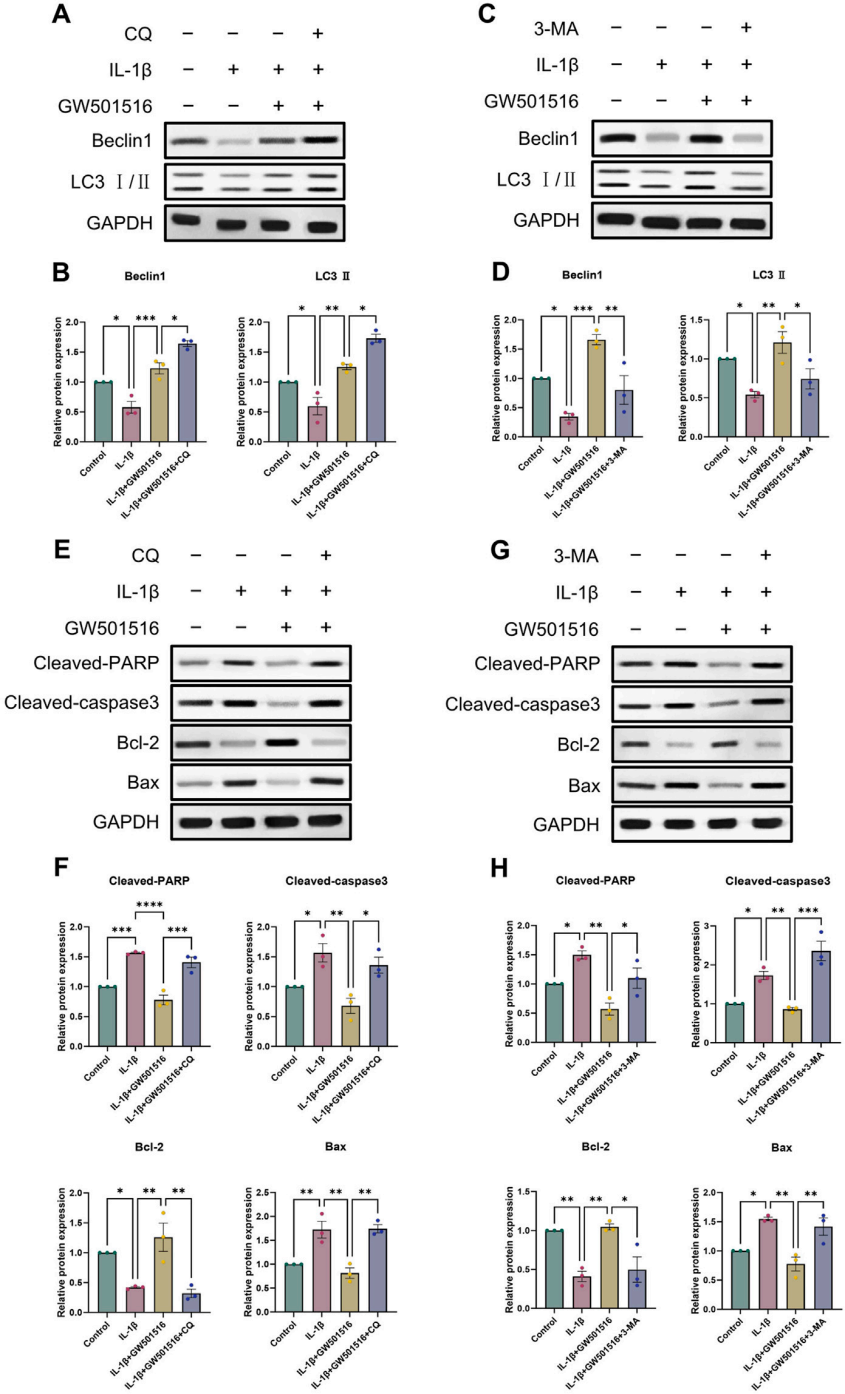
PPARδ activation increases the expression of genes associated to autophagy and reduces apoptosis in chondrocytes. **(A–D)** Chondrocytes were exposed to IL-1β, GW501516, CQ, 3-MA, Western blot as well as quantitative assessment the expression of Beclin1 and LC3 Ⅱ using GAPDH as an endogenous reference. **(E–H)** Chondrocytes were exposed to IL-1β, GW501516, CQ, 3-MA, Western blot as well as quantitative assessment the expression of Cleaved-PARP, Cleaved-caspase3, Bcl-2 and Bax using GAPDH as an endogenous reference. All data are expressed as mean ± SD (*n* = 3); *p < 0.05, **p < 0.01, ***p < 0.001.

Given that we found a correlation between PPARδ and autophagy, we analyzed *in vitro* by Western blot whether PPARδ regulates chondrocyte apoptosis through autophagic activity. We selected 3-methyladenine (3-MA) and chloroquine (CQ) for this part. 3-MA inhibits PI3K, which in turn inhibits autophagy by preventing the production of autophagic vesicles. CQ hampered the autophagosome’s internal degradation process and prevented autophagic vesicles from fusing with lysosomes. Autophagic vesicles kept building up because the cells were still attempting to fuse them with lysosomes, which led to “autophagic stagnation” (Mizushima et al., 2010; Pasquier, 2015; Galluzzi et al., 2017). We first observed that the use of 3-MA reversed the rescuing impact of PPARδ activation upon autophagic loss, which eventually led to the downregulation of the expression of autophagic markers (Beclin1, LC3 Ⅱ) (Figures 7C, D). However, the rescue effect of PPARδ activation on autophagy loss was further enhanced after the use of autophagy inhibitor chloroquine (CQ), and the expression of autophagy markers (Beclin1, LC3 Ⅱ) was further upregulated (Figures 7A, B). The above results again demonstrated the regulatory effect of PPARδ activation on autophagic activity. Furthermore, it was discovered that the use of 3-MA and CQ again greatly reversed the decrease in chondrocyte apoptosis induced by PPARδ activation (Figures 7E–H). In conclusion, our findings imply that Chondrocyte protection afforded by PPARδ is partially attributed to autophagic action.

### 3.6 PPARδ induces autophagy through AKT/mTOR signaling pathway

**FIGURE 8:**
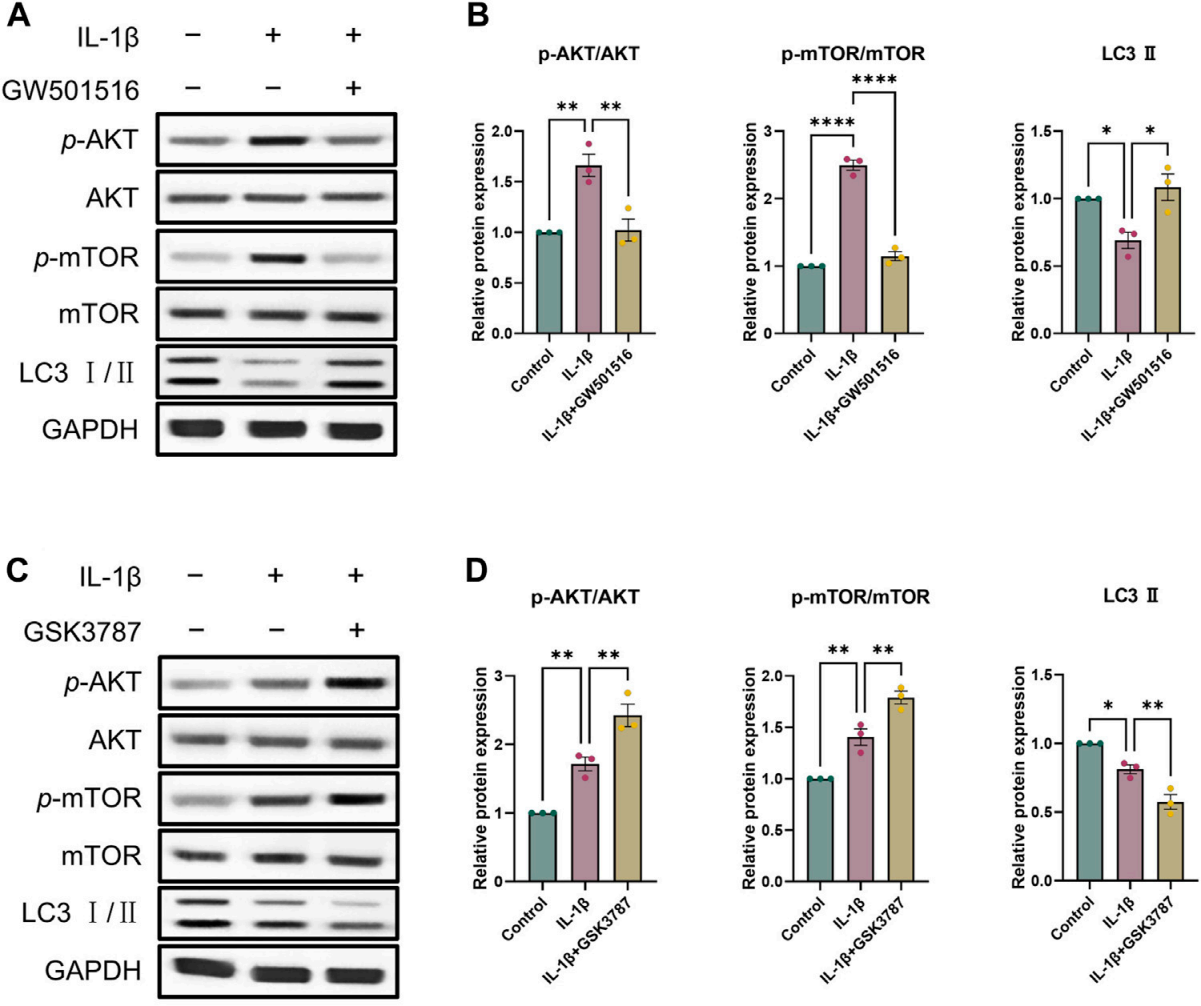
PPARδ induces autophagy through AKT/mTOR signaling pathway. **(A, B)** After the activation of PPARδ, the expression of p-AKT, AKT, p-mTOR, mTOR and LC3 II was quantitatively assessed using ImageJ with GAPDH as an endogenous reference. **(C, D)** After inhibition of PPARδ, the expression of p-AKT, AKT, p-mTOR, mTOR and LC3 II was quantitatively assessed using ImageJ with GAPDH as an endogenous reference. All data are expressed as mean ± SD (*n* = 3); *p < 0.05, **p < 0.01, ****p < 0.0001.

As a crucial pathway for autophagy, AKT/mTOR signaling is one that we looked at in order to learn more about the precise molecular processes by which PPARδ controls autophagy (Kim and Guan, 2015). To investigate whether PPARδ mediates autophagy via AKT/mTOR signaling, we activated PPARδ of chondrocytes, and the results showed that p-AKT and p-mTOR expression dramatically enhanced after the treatment of IL-1β, and LC3 Ⅱ expression decreased significantly; while After PPARδ activation, p-AKT and p-mTOR that were raised in response to IL-1β were suppressed and LC3 Ⅱ expression was upregulated (Figures 8A, B). In addition, we applied PPARδ antagonist to antagonize PPARδ, which again confirmed our findings that PPARδ antagonism significantly increased IL-1β-induced p-AKT and p-mTOR activation (Figures 8C, D). Taken together, PPARδ activation promoted autophagy in rat chondrocytes by blocking the activation of AKT/mTOR signaling generated by IL-1β.

### 3.7 PPARδ activation attenuates OA-induced joint injury in rats

**FIGURE 9:**
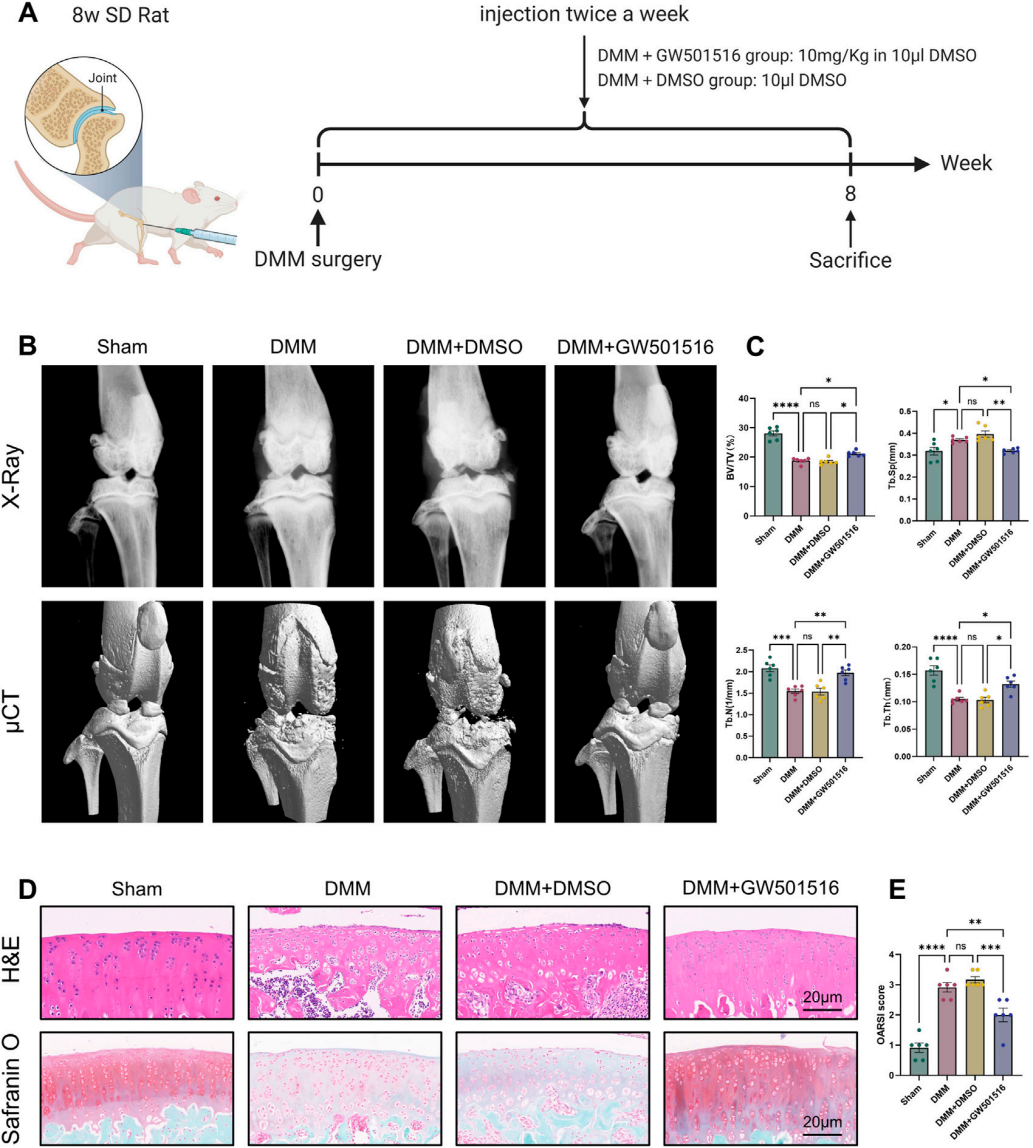
PPARδ activation attenuates OA-induced joint injury in rats. **(A)** Timeline of rat experiments and their associated interventions. The chart drawn with BioRender.com. **(B)** Osteophytes was assessed by X-ray and micro-CT. **(C)** Quantitative analysis of microcomputed tomography: BV/TV, percent bone volume; Tb.N, trabecular number; Tb.Th, trabecular thickness; Tb. Sp, trabecular separation **(D)** H&E staining, Safranine O staining (bar:20 μm). **(E)** The OARSI score. All data are expressed as mean ± SD (*n* = 6); *p < 0.05, **p < 0.01, ***p < 0.001, ****p < 0.0001.

To look into PPARδ′s potential protective role against OA *in vivo*, we constructed rat OA model and injected PPARδ agonist into rats on postoperative day 2 to assess the therapeutic effect of PPARδ activation. The figure shows the illustration and schedule of all relevant interventions (Figure 9A). After tissue collection 8 weeks postoperatively, the rat knee specimens were subjected to X-ray, micro-CT, H&E staining, Safranin O staining and OARSI scoring to assess the differences in histological morphology. The results showed that intra-articular activation of PPARδ in the joint cavity of rats decreased osteophytes compared with the DMM + DMSO group (Figure 9B). Furthermore, micro-CT data demonstrated that, in comparison to DMM + DMSO group, PPARδ activation improved BV/TV, Tb. N, and Tb. Th, and reduced Tb. Sp (Figure 9C). Results of HE and SO staining also indicated that in comparison to DMM + DMSO group, there was less damage to the articular cartilage surface, a healthier cartilage surface, and more abundant proteoglycans following PPARδ activation. Furthermore, in comparison with DMM and DMM + DMSO groups, our study demonstrated that PPARδ activation significantly lowered the OARSI score (Figures 9D,E).

**FIGURE 10:**
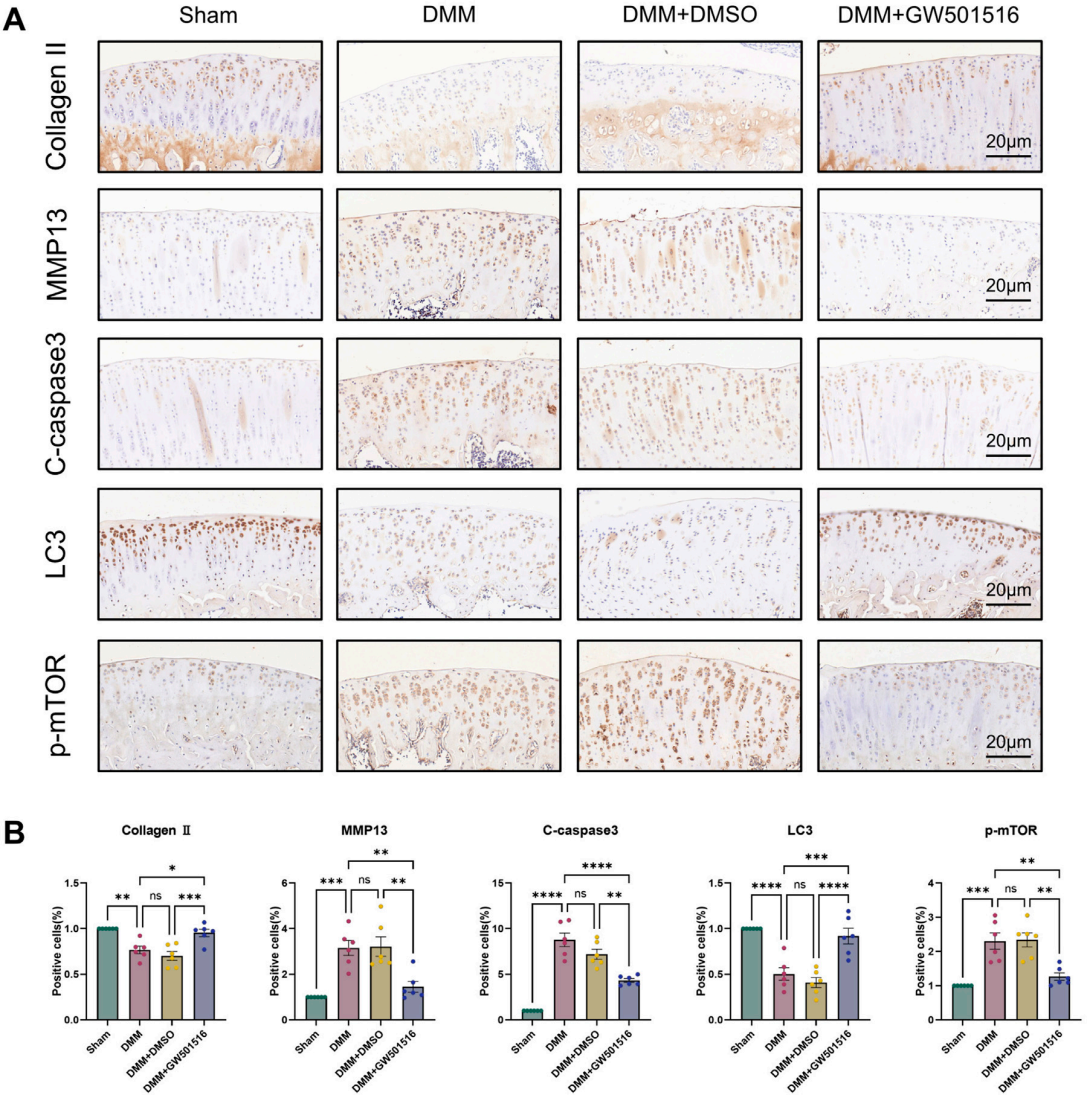
PPARδ activation ameliorates ECM degradation and apoptosis through autophagy induced by AKT/mTOR signaling pathway. **(A)** The expression of Collagen II, MMP13, Cleaved-caspase3, LC3 and p-mTOR in rat articular cartilage was assessed by immunohistochemistry, and **(B)** related quantitative analysis of the proportion of immunohistochemistry-positive cells. All data are expressed as mean ± SD (*n* = 6); *p < 0.05, **p < 0.01, ***p < 0.001, ****p < 0.0001.

**FIGURE 11:**
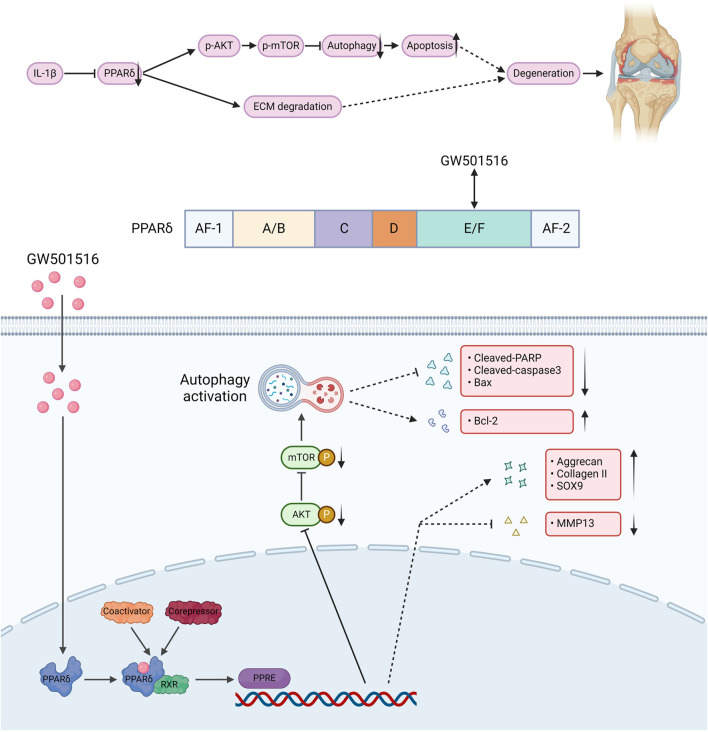
The structure of PPARδ and the site of action of GW501516. GW501516 attenuated ECM degradation through activation of PPARδ, In addition, PPARδ activation inhibits AKT/mTOR phosphorylation, which in turn activates autophagy and attenuates apoptosis, ultimately improving cartilage degeneration. A schematic chart drawn with BioRender.com.

We also found that *in vivo* PPARδ activation in rats improved cartilage ECM catabolism and apoptosis, and increased the expression of LC3. Furthermore, we performed *in vivo* p-mTOR immunohistochemical staining, which further confirmed that PPARδ *in vitro* regulates autophagy via the AKT/mTOR signaling (Figures 10A, B). Finally, we provide a schematic diagram to show how PPARδ affects OA and possible modes of action (Figure 11). All of these findings point to the possibility that PPARδ promotes autophagy through the AKT/mTOR signaling to alleviate OA-induced articular cartilage damage in rats. Therefore, PPARδ might be a viable OA treatment target.

## 4 Discussion

Up to now, the pathogenesis of OA is complex and involves the regulation of multiple pathways and phenotypes, such as anabolic and catabolic metabolism, apoptosis, and autophagy (Hwang and Kim, 2015; Rahmati et al., 2017). Therefore, its pathogenesis remains to be further elucidated. In this study, we found that PPARδ expression was reduced in OA chondrocytes, and there was a time-dependent and dose-dependent relationship between its expression and IL-1β. Therefore, it can be used to predict the severity of OA, especially in terms of Kellgren-Lawrence grading. Inflammatory factors, including TGF-β and IL-1β, are essential to the pathophysiology of OA. PPARδ activation effectively enhances Aggrecan and Collagen II expression, and decreases MMP13 expression of OA cartilage ECM, while PPARδ antagonism exhibits the opposite effect. In addition, apoptosis is the prominent feature of OA, and our study found that PPARδ activation drives increased expression of Bcl-2 and leads to decreased expression of Cleaved-PARP, Cleaved-caspase3, and Bax. However, PPARδ antagonism led to further exacerbation of IL-1β-induced apoptosis. The above study showed that PPARδ activation partially inhibited chondrocyte apoptosis. However, we still need to further explore the complex mechanisms by which PPARδ regulates cartilage degeneration.

Autophagy maintains intracellular homeostasis in chondrocytes and removes degenerated and damaged organelles as well as proteins (Glick et al., 2010). In response to stimuli such as inflammation, aging, and oxidative stress, autophagy can maintain normal cell renewal and metabolism and rescue apoptosis (Mizushima et al., 2008; Mizushima and Komatsu, 2011; Guan et al., 2013; Kim and Lee, 2014; Klionsky et al., 2021; Li et al., 2021). Autophagy has been widely reported to be involved in the development of tumors (Levy et al., 2017), diabetes (Sakai et al., 2019; Dewanjee et al., 2021), cardiovascular diseases (Shirakabe et al., 2016; Sciarretta et al., 2018), and OA (Yang et al., 2020; Chen et al., 2022). Therefore, we hypothesized that there is a relationship between PPARδ and autophagy. The study demonstrated that PPARδ does have an association with autophagy. It was observed that PPARδ antagonism reduced autophagy proteins expression in chondrocytes, whereas PPARδ activation increased them. In addition, we demonstrated that PPARδ regulates chondrocyte apoptosis through autophagy.

To probe the detailed molecular mechanism of PPARδ regulation of cartilage degeneration, we considered that PPARδ activation activates autophagy by the AKT/mTOR signaling. The primary regulator of chondrocyte autophagy is AKT/mTOR signaling (Kuma et al., 2017). Moreover, mTORC1 primarily regulates autophagy negatively and is subject to regulation via many molecules that impact autophagic activity (Yang et al., 2020). As OA worsens, mTOR upregulates and mediates the inhibition of chondrocyte autophagy, which finally causes cartilage degradation by reducing the beneficial impact on cartilage. The OA models showed upregulation of autophagy and decreased levels of apoptosis after mTOR knockdown (Zheng et al., 2021). On the contrary, inhibition of autophagy by rapamycin and inhibition of apoptosis by mTORC1 showed a significant alleviating effect on OA. In DMM mice model, intra-articular injection of the autophagy inducer resveratrol to activate chondrocyte autophagy significantly played a protective role against degenerating cartilage (Qin et al., 2017). In this work, we found that PPARδ activation prevented IL-1β-induced AKT/mTOR signaling activation, which in turn activated autophagy and attenuated chondrocyte apoptosis. In contrast, PPARδ antagonism presented the opposite situation.

The study has some limitations. First, we only studied male SD rats, and the beneficial impact of PPARδ activation on chondrocytes should be confirmed in female, obese, aging-related OA models. Second, we used only the agonist and inhibitor of PPARδ, and the use of overexpressed lentiviral articular cavity injections or the construction of transgenic rats would further corroborate our conclusions. Third, PPARδ expression in human chondrocytes was not investigated by us. The similarity between rat articular chondrocytes and human articular chondrocytes is minimal. To further confirm the results of the study, the specific molecular mechanisms between PPARδ and OA should be investigated using human articular chondrocytes.

In conclusion, we showed that PPARδ activates autophagy through inhibiting AKT/mTOR signaling pathway, reduced chondrocyte apoptosis and ECM degradation, and finally exerts a protective effect against OA.

## Data Availability

The original contributions presented in the study are included in the article/Supplementary Materials, further inquiries can be directed to the corresponding authors.

## References

[B1] Abramoff B. Caldera F. E. (2020). Osteoarthritis: pathology, diagnosis, and treatment options. Med. Clin. North Am. 104, 293–311. 10.1016/j.mcna.2019.10.007 32035570

[B2] Ansari M. Y. Ahmad N. Haqqi T. M. (2020). Oxidative stress and inflammation in osteoarthritis pathogenesis: role of polyphenols. Biomed. Pharmacother. 129, 110452. 10.1016/j.biopha.2020.110452 32768946 PMC8404686

[B3] Bouderlique T. Vuppalapati K. K. Newton P. T. Li L. Barenius B. Chagin A. S. (2016). Targeted deletion of Atg5 in chondrocytes promotes age-related osteoarthritis. Ann. Rheumatic Dis. 75, 627–631. 10.1136/annrheumdis-2015-207742 PMC4789686 26438374

[B4] Chen X. Gong W. Shao X. Shi T. Zhang L. Dong J. (2022). METTL3-mediated m(6)A modification of ATG7 regulates autophagy-GATA4 axis to promote cellular senescence and osteoarthritis progression. Ann. Rheum. Dis. 81, 87–99. 10.1136/annrheumdis-2021-221091 34706873

[B5] Christofides A. Konstantinidou E. Jani C. Boussiotis V. A. (2021). The role of peroxisome proliferator-activated receptors (PPAR) in immune responses. Metabolism 114, 154338. 10.1016/j.metabol.2020.154338 32791172 PMC7736084

[B6] Corrales P. Izquierdo-Lahuerta A. Medina-Gomez G. (2018). Maintenance of kidney metabolic homeostasis by PPAR gamma. Int. J. Mol. Sci. 19, 2063. 10.3390/ijms19072063 30012954 PMC6073436

[B7] Creamer P. Hochberg M. C. (1997). Osteoarthritis. Lancet 350, 503–508. 10.1016/S0140-6736(97)07226-7 9274595

[B8] Dewanjee S. Vallamkondu J. Kalra R. S. John A. Reddy P. H. Kandimalla R. (2021). Autophagy in the diabetic heart: a potential pharmacotherapeutic target in diabetic cardiomyopathy. Ageing Res. Rev. 68, 101338. 10.1016/j.arr.2021.101338 33838320

[B9] Dubois V. Eeckhoute J. Lefebvre P. Staels B. (2017). Distinct but complementary contributions of PPAR isotypes to energy homeostasis. J. Clin. Invest. 127, 1202–1214. 10.1172/JCI88894 28368286 PMC5373878

[B10] Galluzzi L. Bravo-San Pedro J. M. Levine B. Green D. R. Kroemer G. (2017). Pharmacological modulation of autophagy: therapeutic potential and persisting obstacles. Nat. Rev. Drug Discov. 16, 487–511. 10.1038/nrd.2017.22 28529316 PMC5713640

[B11] Glick D. Barth S. Macleod K. F. (2010). Autophagy: cellular and molecular mechanisms. J. Pathol. 221, 3–12. 10.1002/path.2697 20225336 PMC2990190

[B12] Glyn-Jones S. Palmer A. J. Agricola R. Price A. J. Vincent T. L. Weinans H. (2015). Osteoarthritis. Lancet 386, 376–387. 10.1016/S0140-6736(14)60802-3 25748615

[B13] Gross B. Pawlak M. Lefebvre P. Staels B. (2017). PPARs in obesity-induced T2DM, dyslipidaemia and NAFLD. Nat. Rev. Endocrinol. 13, 36–49. 10.1038/nrendo.2016.135 27636730

[B14] Guan J. L. Simon A. K. Prescott M. Menendez J. A. Liu F. Wang F. (2013). Autophagy in stem cells. Autophagy 9, 830–849. 10.4161/auto.24132 23486312 PMC3672294

[B15] Heck B. E. Park J. J. Makani V. Kim E. C. Kim D. H. (2017). PPAR-Delta agonist with mesenchymal stem cells induces type II collagen-producing chondrocytes in human arthritic synovial fluid. Cell Transpl. 26, 1405–1417. 10.1177/0963689717720278 PMC5680970 28901183

[B16] Hosseinzadeh A. Kamrava S. K. Joghataei M. T. Darabi R. Shakeri-Zadeh A. Shahriari M. (2016). Apoptosis signaling pathways in osteoarthritis and possible protective role of melatonin. J. Pineal Res. 61, 411–425. 10.1111/jpi.12362 27555371

[B17] Hwang H. S. Kim H. A. (2015). Chondrocyte apoptosis in the pathogenesis of osteoarthritis. Int. J. Mol. Sci. 16, 26035–26054. 10.3390/ijms161125943 26528972 PMC4661802

[B18] Kim K. H. Lee M.-S. (2014). Autophagy—a key player in cellular and body metabolism. Nat. Rev. Endocrinol. 10, 322–337. 10.1038/nrendo.2014.35 24663220

[B19] Kim Y. C. Guan K. L. (2015). mTOR: a pharmacologic target for autophagy regulation. J. Clin. Invest. 125, 25–32. 10.1172/JCI73939 25654547 PMC4382265

[B20] Klionsky D. J. Petroni G. Amaravadi R. K. Baehrecke E. H. Ballabio A. Boya P. (2021). Autophagy in major human diseases. EMBO J. 40, e108863. 10.15252/embj.2021108863 34459017 PMC8488577

[B21] Kuma A. Komatsu M. Mizushima N. (2017). Autophagy-monitoring and autophagy-deficient mice. Autophagy 13, 1619–1628. 10.1080/15548627.2017.1343770 28820286 PMC5640176

[B22] Levy J. M. M. Towers C. G. Thorburn A. (2017). Targeting autophagy in cancer. Nat. Rev. Cancer 17, 528–542. 10.1038/nrc.2017.53 28751651 PMC5975367

[B23] Li W. He P. Huang Y. Li Y. F. Lu J. Li M. (2021). Selective autophagy of intracellular organelles: recent research advances. Theranostics 11, 222–256. 10.7150/thno.49860 33391472 PMC7681076

[B24] Liao S. Zheng Q. Shen H. Yang G. Xu Y. Zhang X. (2023). HECTD1‐Mediated ubiquitination and degradation of Rubicon regulates autophagy and osteoarthritis pathogenesis. Arthritis and Rheumatology 75, 387–400. 10.1002/art.42369 36121967

[B25] Liu Y. Luo S. Zhan Y. Wang J. Zhao R. Li Y. (2020). Increased expression of PPAR-gamma modulates monocytes into a M2-like phenotype in SLE patients: an implicative protective mechanism and potential therapeutic strategy of systemic lupus erythematosus. Front. Immunol. 11, 579372. 10.3389/fimmu.2020.579372 33584646 PMC7873911

[B26] Michalik L. Wahli W. (2006). Involvement of PPAR nuclear receptors in tissue injury and wound repair. J. Clin. Invest. 116, 598–606. 10.1172/JCI27958 16511592 PMC1386118

[B27] Mirza A. Z. Althagafi Ii Shamshad H. (2019). Role of PPAR receptor in different diseases and their ligands: physiological importance and clinical implications. Eur. J. Med. Chem. 166, 502–513. 10.1016/j.ejmech.2019.01.067 30739829

[B28] Mizushima N. Komatsu M. (2011). Autophagy: renovation of cells and tissues. Cell 147, 728–741. 10.1016/j.cell.2011.10.026 22078875

[B29] Mizushima N. Levine B. Cuervo A. M. Klionsky D. J. (2008). Autophagy fights disease through cellular self-digestion. Nature 451, 1069–1075. 10.1038/nature06639 18305538 PMC2670399

[B30] Mizushima N. Yoshimori T. Levine B. (2010). Methods in mammalian autophagy research. Cell 140, 313–326. 10.1016/j.cell.2010.01.028 20144757 PMC2852113

[B31] Muller R. (2017). PPARβ/δ in human cancer. Biochimie 136, 90–99. 10.1016/j.biochi.2016.10.019 27916645

[B32] Palomer X. Capdevila-Busquets E. Botteri G. Salvado L. Barroso E. Davidson M. M. (2014). PPARβ/δ attenuates palmitate-induced endoplasmic reticulum stress and induces autophagic markers in human cardiac cells. Int. J. Cardiol. 174, 110–118. 10.1016/j.ijcard.2014.03.176 24767130

[B33] Pasquier B. (2015). Autophagy inhibitors. Cell. Mol. Life Sci. 73, 985–1001. 10.1007/s00018-015-2104-y 26658914 PMC11108294

[B34] Poleni P. E. Bianchi A. Etienne S. Koufany M. Sebillaud S. Netter P. (2007). Agonists of peroxisome proliferators-activated receptors (PPAR) alpha, beta/delta or gamma reduce transforming growth factor (TGF)-beta-induced proteoglycans' production in chondrocytes. Osteoarthr. Cartil. 15, 493–505. 10.1016/j.joca.2006.10.009 17140817

[B35] Qin N. Wei L. Li W. Yang W. Cai L. Qian Z. (2017). Local intra-articular injection of resveratrol delays cartilage degeneration in C57BL/6 mice by inducing autophagy via AMPK/mTOR pathway. J. Pharmacol. Sci. 134, 166–174. 10.1016/j.jphs.2017.06.002 28669597

[B36] Rahmati M. Nalesso G. Mobasheri A. Mozafari M. (2017). Aging and osteoarthritis: central role of the extracellular matrix. Ageing Res. Rev. 40, 20–30. 10.1016/j.arr.2017.07.004 28774716

[B37] Sakai S. Yamamoto T. Takabatake Y. Takahashi A. Namba-Hamano T. Minami S. (2019). Proximal tubule autophagy differs in type 1 and 2 diabetes. J. Am. Soc. Nephrol. 30, 929–945. 10.1681/asn.2018100983 31040190 PMC6551771

[B38] Sciarretta S. Maejima Y. Zablocki D. Sadoshima J. (2018). The role of autophagy in the heart. Annu. Rev. Physiology 80, 1–26. 10.1146/annurev-physiol-021317-121427 29068766

[B39] Shearer B. G. Wiethe R. W. Ashe A. Billin A. N. Way J. M. Stanley T. B. (2010). Identification and characterization of 4-chloro-N-(2-{[5-trifluoromethyl-2-pyridyl]sulfonyl}ethyl)benzamide (GSK3787), a selective and irreversible peroxisome proliferator-activated receptor delta (PPARdelta) antagonist. J. Med. Chem. 53, 1857–1861. 10.1021/jm900464j 20128594

[B40] Shirakabe A. Ikeda Y. Sciarretta S. Zablocki D. K. Sadoshima J. (2016). Aging and autophagy in the heart. Circulation Res. 118, 1563–1576. 10.1161/circresaha.116.307474 27174950 PMC4869999

[B41] Song J. Y. Park J. S. Kim J. H. Wang J. H. Heck H. C. Heck B. E. (2022). PPARδ agonist promotes type II cartilage formation in a rabbit osteochondral defect model. Cells 11, 2934. 10.3390/cells11192934 36230897 PMC9564068

[B42] Vasheghani F. Zhang Y. Li Y.-H. Blati M. Fahmi H. Lussier B. (2015). PPARγ deficiency results in severe, accelerated osteoarthritis associated with aberrant mTOR signalling in the articular cartilage. Ann. Rheumatic Dis. 74, 569–578. 10.1136/annrheumdis-2014-205743 PMC4345902 25573665

[B43] Wagner N. Wagner K. D. (2020). The role of PPARs in disease. Cells 9, 2367. 10.3390/cells9112367 33126411 PMC7692109

[B44] Wood G. Neilson J. Cottrell E. Hoole S. P. Guideline C. (2023). Osteoarthritis in people over 16: diagnosis and management-updated summary of NICE guidance. BMJ 380, 24. 10.1136/bmj.p24 36693668

[B45] Yang H. Wen Y. Zhang M. Liu Q. Zhang H. Zhang J. (2020). MTORC1 coordinates the autophagy and apoptosis signaling in articular chondrocytes in osteoarthritic temporomandibular joint. Autophagy 16, 271–288. 10.1080/15548627.2019.1606647 31007149 PMC6984599

[B46] Zheng L. Zhang Z. Sheng P. Mobasheri A. (2021). The role of metabolism in chondrocyte dysfunction and the progression of osteoarthritis. Ageing Res. Rev. 66, 101249. 10.1016/j.arr.2020.101249 33383189

[B47] Zhou Y. Chen X. Qu N. Zhang B. Xia C. (2019). Chondroprotection of PPARα activation by WY14643 via autophagy involving Akt and ERK in LPS-treated mouse chondrocytes and osteoarthritis model. J. Cell Mol. Med. 23, 2782–2793. 10.1111/jcmm.14184 30729704 PMC6433667

