# Supplementary figures and images for: PPARδ agonist protects against osteoarthritis by activating AKT/mTOR signaling pathway-mediated autophagy
Source: Front Pharmacol. 2024 Mar 21;15:1336282. doi: 10.3389/fphar.2024.1336282 (PMC10991777; doi:10.3389/fphar.2024.1336282)

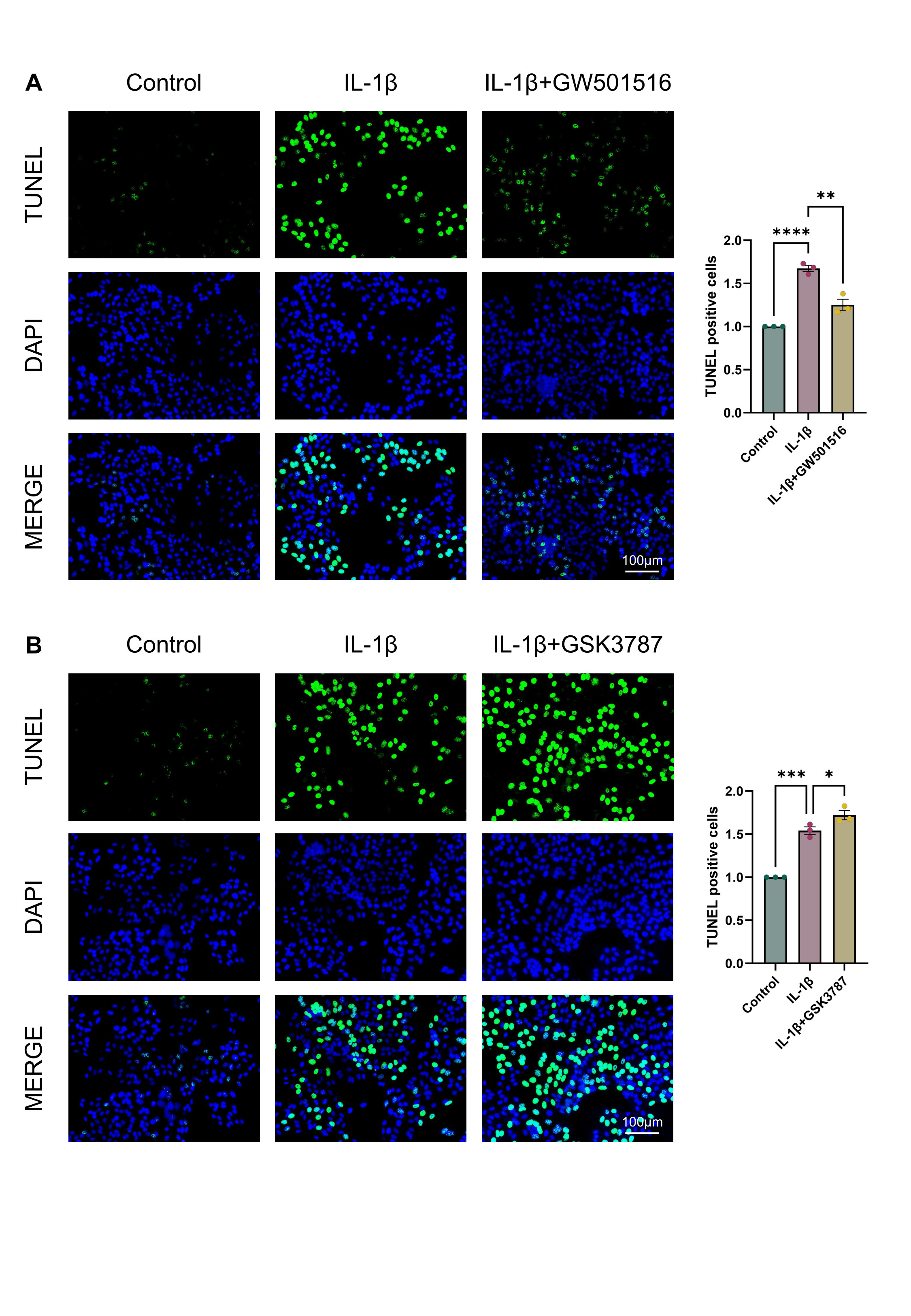

Supplement: Supplementary file 1 [file Image2.tif]
